# The Red flag! risk assessment among medical homeopaths in Norway: a qualitative study

**DOI:** 10.1186/1472-6882-12-150

**Published:** 2012-09-11

**Authors:** Trine Stub, Terje Alræk, Anita Salamonsen

**Affiliations:** 1 Department of Community Medicine, NAFKAM (The National Research Center in Complementary and Alternative medicine), University of Tromsø, Tromsø, 9037, Norway

**Keywords:** Homeopathy, Conventional medicine, Homeopathic aggravations, Adverse effects, Medical homeopaths, Patient safety, Risk assessment

## Abstract

**Background:**

Homeopathy is widely used, and many European physicians practice homeopathy in addition to conventional medicine. Adverse effects in homeopathy are not expected by homeopaths due to the negligible quantities of active substances in a remedy. However, we questioned if homeopathic aggravation, which is described as a temporary worsening of existing symptoms following a correct homeopathic remedy, should be regarded as adverse effects or ruled out as desirable events of the treatment. In order to improve knowledge in an unexplored area of patient safety, we explored how medical homeopath discriminate between homeopathic aggravations and adverse effects, and how they assessed patient safety in medical practice.

**Method:**

A qualitative approach was employed using focus group interviews. Two interviews with seven medical homeopaths were performed in Oslo, Norway. The participants practiced homeopathy besides conventional medicine. Qualitative content analysis was used to analyze the text data. The codes were defined before and during the data analysis.

**Results:**

According to the medical homeopaths, a feeling of well-being may be a criterion to distinguish homeopathic aggravations from adverse effects. There was disagreement among the participants whether or not homeopathic treatment produced adverse effects. However, they agreed when an incorrect remedy was administrated, it may create a disruption or suppressive reaction in the patient. This was not perceived as adverse effects but a possibility to prescribe a new remedy as new symptoms emerge. This study revealed several advantages for the patients as the medical homeopaths looked for dangerous symptoms which may enhance safety. The patient was given time and space, which enabled the practitioner to see the complete picture. A more comprehensive toolkit gave the medical homeopaths a feeling of professionalism.

**Conclusion:**

This explorative study investigated how Medical Homeopaths understood and assessed risk in their clinical practice. A feeling of well-being emerging soon after taking the remedy was the most important criterion for discriminating between Homeopathic Aggravations and Adverse Effects in clinical practice. The Medical Homeopaths used the view of both professions and always looked for red flag situations in the consultation room. They combined knowledge from two treatment systems which may have advantages for the patient. These tentative results deserve further research efforts to improve patient safety among users of homeopathy. For further research we find it important to improve and develop concepts that are unique to homeopathy in order to validate and modernize this medical practice.

## Background

Homeopathy is practiced worldwide and is a treatment system based on two principles: i) the law of Similaris (similia similibus curentur), meaning “like cures like” and ii) individualization [1,2]. The hypothesis of the Law of Similaris is that substances capable of causing certain symptoms in healthy subjects can be used to cure people who suffer from similar symptoms. Homeopathic medicines are undergoing a process of stepwise dilution and vigorous shaking [3,4]. Some of these dilutions are known to be “ultra-molecular”, indicating that they are diluted to such a degree that not even a single molecule of the original substance is left in the remedy. Individualization is understood as the use of the patient’s individual characteristics when deciding which homeopathic remedy to prescribe. Thus, patients may get different remedies for the same health problem [2].

### Safety of homeopathic remedies

It is a widespread belief that homeopathy is natural and therefore safe, and that people can be treated without Adverse Effects (AE) [5-7]. AE from homeopathy may not be expected due to its mode of influencing body’s self-regulation, and the negligible quantities of active substance in a remedy [8]. In a systematic review [9] the authors concluded that homeopathic medicines may provoke AE, but these are generally mild and transient. Under-reporting may be the reason for the lack of information about safety in homeopathy [10,11]. Moreover, an absence of reports of AE does not mean that they do not occur [8,12].

### Homeopathic aggravation

Homeopathic Aggravation (HA) is a temporary worsening of existing symptoms following the administration of a correct homeopathic remedy and is usually followed by an improvement [13-15]. It indicates that the individual is responding to the medication by generating or increasing symptoms, which is seen as the body’s way of coping with illness and part of the healing process [2,16]. A question about safety is whether HA that, according to the homeopaths, often occurs in homeopathic treatment should be considered as AE or ruled out as desirable and positive events in the course of homeopathic treatment [6]. Conventional health care providers do not distinguish between AE and aggravation, as they see these events as similar.

### Risk assessment in homeopathic treatment

Some authors have suggested that homeopathy itself may be considered free of direct risk, whereas it may be associated with indirect risks, related to the prescriber rather than the medicine [11,17]. Homeopathic practice in Norway (as well as in the United Kingdom (UK)) is currently unregulated. Anybody, irrespective of training or registration can practice homeopathy. This has been described as the main source of risk [11]. Another indirect risk situation is when a homeopath without medical training prescribes homeopathy when a conventional treatment is more appropriate [18]. A similar situation is a delay of meaningful diagnostic or therapeutic measures, meaning that patients with diseases which cannot be cured using homeopathic remedies are treated too late or not at all with conventional medicine [5].

### Risk assessment in conventional medicine

The requirement of doing no harm (nil nocere) comes from Hippocrates. In his first book on epidemics he stated that a doctor needs to keep two things in mind: to do good or to do no harm (nonmaleficence) [19,20]. This principle is one of four in clinical medicine. The others are autonomy, beneficence, and justice [21]. In modern times patient safety is generally understood as preventing and limiting unfortunate consequences or damages due to any health treatment [20,21]. In each case individual judgment is of importance when evaluating whether or not the demand for liability has been met [20,22]. The demand for professional liability is described in paragraph 4 of The Norwegian Medical Personnel Act (Helsepersonelloven). It states that *medical personnel is to perform according to the demands for professional liability and tender care which may be expected based on the qualifications of the personnel, the type of work and the situation*[20]*.*

Adverse effects are regularly observed in clinical practice [23]. However, there is a culture for under-reporting such events [20]. One study showed that adverse drug events occur in 25% of primary care patients, and that 11% of these events were preventable [24]. Weingart [25] found that disability occurred in 3.7% of the patients who had been admitted to acute care hospitals at the time of discharge. Moreover, missed or delayed diagnoses are the most common problem leading to malpractice and claims in the outpatient setting [26]. As individual judgment is an important factor when evaluating and handling risk in health care situations, it is ethically important to investigate how this is done in homeopathic clinical practices.

### Medical homeopaths

France, the UK and Norway are countries in which the number of homeopathic practitioners varies greatly. In France where there are 60,000 GPs, more than 5,000 (8%) are classified as Medical Homeopaths (MH) [27]. In the UK there are 1,000 MHs registered with the Faculty of Homeopathy, and about 2,200 non-medical homeopaths with one of the professional bodies [27], and homeopathy is available from the National Health Services. In Norway there are 20 MHs, which is 0.0008% of the total 26,000 GPs [28]. Norwegian MHs may get their homeopathic certification from two private colleges, including a five or three year part time program. In addition there are 230 non-medical homeopaths registered with the Norwegian Association of Homeopaths. This shows that Norwegian MHs are an exclusive group of practitioners compared to France and the UK.

As MHs belong to these two different medical treatment systems, we wanted to ask them how they assess safety for their patients.

### Research questions

In order to improve knowledge in an underexplored area of safety, we wanted to investigate when an initial HA becomes AE and when it ought to be reported as such. In a previous study with eleven experienced classical homeopaths we found that HA was perceived to be a subtle and multifaceted event and highly skilled homeopaths were required to identify and report HA. The participants defined AE as *“undesirable effects of a remedy”*, as this definition is pragmatic, flexible and more in line with the holistic paradigm that the homeopaths represent. Eight criteria that distinguish HA from AE were identified. These criteria may enhance patient safety as they support practitioners in identifying an undesirable effect of a remedy [29].

In this study we wanted to explore how MHs discriminate between HA and AE in their practice. Thus, the research questions were: 1. How do the MHs understand and discriminate between HA and AE in their clinical practice? 2. How do the MHs assess patient safety in their medical practice?

## Methods

### Design

A qualitative approach was employed using focus group interviews [30], as qualitative studies may contribute to a deeper understanding and thorough knowledge of important issues in health and well-being, especially in situations in which we have limited previous knowledge of our phenomenon of interest [31,32]. Group interviews were chosen over individual interviews as the dialogue between the participants would reveal diversity of the relevant aspects of interest. In the field of CAM the validity of qualitative methodology has been identified as fundamental to understanding and describing the philosophical basis, key treatment components and contextual frameworks of CAM modalities [33,34]. This study has been approved by the regional Ethics Committee for Medical and Health Science in North Norway, and meets the standard of The Helsinki Declaration in its revised version of 1975 and its amendments of 1983, 1989 and 1996.

### Participants

We wanted to include medical doctors who also had homeopathic certification. With the help from the staff at the Norwegian Academy of Natural medicine (NAN) we identified 20 medical homeopaths who were asked by phone to participate. Seven had left the field and six did not answer our calls. Four women and three men accepted the invitation. They all initiated their homeopathic training during or shortly after graduating from medical school. Their reason for starting practicing homeopathy was a successful personal or close relative’s experience with the therapy. They had practiced classical homeopathy over a period of 4–35 years with an average of 16 years and conventional medicine over a period of 10–35 years with an average of 22 years. The first homeopathic consultation varied from 30–90 minutes and they offered a range of additional therapies such as acupuncture, mindfulness, client-centered therapy and magnetic field therapy.

Table 1: Sample characteristics.

**Table 1:** Sample Characteristics

***Focus Group Interview of 7 Medical Homepaths***			
	*Full time hospital doctor (retired)*	*1*	
	*Part time hospital doctor and part time GP with municipality agreement*	*1*	
	*Full time GP with municipality agreement*	*2*	
	*Full time GP without municipality agreement*	*1*	
	*Part time GP without municipality medicine*	*2*	
	*Practicing homeopathy in addition to conventional medicine*	*7*	
*Years of practice*		*Homeopathy*	*Conventional*
	*0*–*4 years*	*2*	*0*
	*5*–*9 years*	*0*	*0*
	*10*–*14 years*	*1*	*2*
	*15*–*19 years*	*2*	*2*
	*20*–*24 years*	*0*	*0*
	*25*–*29 years*	*1*	*1*
	*30*–*35 years*	*1*	*2*
*Classical homeopathy*		*6*	
*Complex and classical homeopathy*		*1*	
*Length of initial homeopathic consultation*			
	*60 minutes*	*2*	
	*90 minutes*	*3*	
	*Between 30 to 90 minutes*	*1*	
	*Between 60 to 90 minutes*	*1*	
*Additonal therapies*			
	*Classic Acupunture*	*1*	
	*Vitamin/mineral/herb*	*1*	
	*Client-Centered therapy (Carl Rogers)*	*1*	
	*Mindfulness/meditation*	*1*	
	*Laying on hands/Healing*	*1*	
	*Nutritional theraphy*	*1*	
	*Magnetic field theraphy*	*1*	
	*Physical relaxation therapy*	*1*	
*Female*		*4*	
*Male*		*3*	

### Focus group interviews

Focus group interviews were chosen as this method is particularly suited to study attitudes and experiences concerning specific topics of which we have little previous knowledge [30,35,36]. Two interviews were conducted in Oslo, Norway with four and three participants respectively. The interviews took place in a private health clinic. They were tape recorded and conducted using an open ended and semi-structured technique [30] with T.S. as the moderator and T.A. and A.S. as observers. The main topics were homeopathic aggravations, adverse effects, risk assessment and the advantages and disadvantages belonging to the two medical paradigms.

### Interview guide

A systematic review of the literature (will be published elsewhere), in which fifty-seven studies were included, formed the basis for the interview guide. These studies provided a systematic description of HA and AE, including how frequently these were reported in the scientific literature. In addition medical and homeopathic literature were searched for information about HA and AE. As the relationship between HA and AE is rather unexplored, definitions of the concepts were sent to the participants in advance as these were used as the fundaments for the interviews. The interview guide and the definitions are available in additional files 1 and 2, respectively.

### Data analysis

We used qualitative content analysis to analyze the transcribed interviews, our text data focusing on the characteristics of language as qualitative communication with attention to the content or contextual meaning. The goal was to provide knowledge and understanding of the phenomena under study through a systematic classification process of coding and identifying themes [30,37,38]. The codes were defined before and during the data analysis. Hence, the coding was a mixed type, using elements from conventional and direct content analysis [38]. From this analysis, concepts and categories were developed. At each stage of the analysis process, the researchers met and discussed after having read the relevant data several times. By this approach the analysis was influenced by the perspective of different professional backgrounds. The answers from the participants were written in Norwegian and the quotations were translated by a native English speaker.

## Results

During the analysis, five main categories were revealed: *homeopathic aggravation, adverse effects, disruption, risk assessment, and benefits of both conventional and homeopathic treatment.*

### Homeopathic aggravation

The MHs claimed that aggravation was something they often observed in clinical practice both as a dramatic and subtle event. One of them expressed:

"Yes, patients tell me when the aggravation is very strong and obvious. However, if the character is more subtle, the initial consultation needs to be rather thorough in order for them and me to understand that some symptoms are so-called initial aggravations."

Another MH added:

"I think it is difficult to determine the exact number of patients who experience initial aggravations. However, I have seen some quite severe cases. Patients suffering from rheumatoid arthritis, eczema and sciatica have got much worse following homeopathic treatment. The illness has exploded. I call it initial aggravations because, as you say, they have more energy and they can handle it."

According to the MHs, the worst aggravation the patients may experience is when the eczema gets worse, as expressed by one of the participants:

"Patients suffering from eczema, who have seen other homeopaths, have come to me. The remedy they had been given was too strong, resulting in a severe aggravation of the eczema. Therefore, dermatologists understand and know that homeopathy works, as they meet such patients [when hospitalized as a result of the homeopathic treatment]."

One participant explained how adults may react when treated with the correct homeopathic remedy:

"If children have felt that nobody loves them, [as adults] they will do everything to be loved, at work and everywhere. This is a terrible condition, and they may experience anger. This is positive [reaction from the remedy], as it is caused by suppressed anger. When the anger appears I say to them “OK, don’t take it out on your partner. You need to understand that this is a very important process for you.”"

According to the MHs, a feeling of well-being (sleeping better and feeling more energetic, mentally and emotionally stronger, and more balanced) is a criterion that may be used to distinguish HA from AE. One MH explained: “The reason why people keep up with these aggravations is that they feel better. There is nothing else to it. You feel better.” These quotations demonstrate that conditions such as eczema and suppressed feelings were most prone to aggravation. A true HA must be followed by an improved sense of well-being. If not, it may be an AE.

### Adverse effects

The participants explained the differences between AE and HA as follows: “When you observe severe aggravations without dynamics [no progression in the patient’s symptoms] that continue over time, I think there is something wrong. You may call it adverse effects.” One female participant said: “I do not like that my patients get depressed. That is a bad sign”. Another stated: “However, I have never seen a serious life threatening event”. A third one expressed:

"To me the distinction between adverse effect and aggravation is defined by the general experience of the patient. If the patient experiences an overall improvement, a general increase in energy, I call the aggravation homeopathic aggravation. If the patient feels generally bad, it is adverse effect. This is the distinction I will make."

One male MH talked about the primary and secondary effect of a remedy, when he referred to AE:

"Hahneman [the founder of homeopathy] claims that medication has two types of reactions. The first is a primary reaction which is the quality of the medication. Then there is the secondary effect, which is equivalent to what is called adverse effects in medicine. That is the body’s reaction to the medication, including all the symptoms that may arise. These adverse effects cause a big problem. I am so happy that I can give my patients remedies which will not cause any adverse effect, as there is no secondary effect of homeopathic remedies. They [the symptoms] disappear and that is why you are cured."

Some of the participants perceived that homeopathy produced AE, described as aggravations with no dynamics, meaning a generally bad feeling without improvement. However, others claimed that there were no AE in homeopathy described as a secondary effect of the remedy. Hence, there were disagreements among the participants whether or not homeopathic treatment produced AE.

### Disruption

A disruptive reaction is a reaction following an incorrect remedy. This causes disappearance of some symptoms and creation of new symptoms, and is frequently seen in clinical practice. In order for this to happen, the patient must be sensitive to the medication. One participant described a personal experience with homeopathy, which was perceived to be a disruptive reaction, as described by one of the participants: “I was extremely depressed over six weeks following a cure of 1 M Sulphur.”

A male participant explained:

"However, this is a good example of an incorrect remedy, but it touches you. So it’s very close. You have had a disruptive reaction. I have no personal relationship to him [the MH], but there was something within him that provoked this reaction. It is too complicated to explain here, but as a therapist you have to consider this."

A female added: “Because he has certain sensitivity to “Sulphur”. Another continued: “However, what you may find from a disruptive reaction is the correct remedy. This gives you a unique possibility to understand, because you know that it [the initial remedy] is incorrect.”When the moderator asked him if he considered this to be AEs, he denied this to be the case.

 These statements demonstrate that when an incorrect remedy is administrated, it may create a disruptive or suppressive reaction in the body. The participants did not perceive this to be AE, but a possibility to prescribe a new remedy as new symptoms emerge. A suppressive reaction is when the symptoms move from a more superficial to a deeper level in the patient, for example when the eczema disappears, and is replaced by asthmatic symptoms.

### Risk assessment

In order to evaluate risk the MHs had to evaluate the patient twice, as one of them explained:

"If you see through the eyes of a doctor you see different things than through the eyes of a homeopath. You look for different things, you ask yourself different questions, and these are two different ways of thinking. It is almost as if you need to think two different thoughts at the same time. These are two different conceptual worlds."

A female explained:

"In my initial consultation there is always a doctor present within me looking for severe symptoms. My way of thinking like a doctor never stops: “Do I ignore something severe, or is this something that needs further examination?”"

Another added:

"It is not difficult to think two thoughts simultaneously. I can easily perform an initial interview as I’m thinking of what remedy is suitable. Sometimes it strikes me: “Are there any red flags here?” I may prescribe a homeopathic remedy and an X-ray requisition to make sure that I do not ignore anything."

The MHs evaluated risk from both a conventional and homeopathic point of view. Based on this, they organized the patient’s symptoms in a twofold manner.

### Benefits of using both conventional and homeopathic treatment

According to the MHs, a reduction of conventional medicine is an advantage for patients visiting them. One female said: “When you start treating a patient on conventional medicine, homeopathy enables you to remove it [the conventional medicine] slowly but surely.” She told about a patient who could reduce her medication for high blood pressure by three-fourths. She explained:

"It is no problem for me that she needs a little bit of her medication. She still needs far less than normal and has fewer adverse effects then she would have had without homeopathic medication. I think it is awesome to have both types of treatment."

The homeopathic consultation allowed the patient to explore her/his entire medical history. According to a MH a new medical diagnosis may be revealed, as the complete picture emerges.

"Sometimes I have diagnosed people who for many years have been visiting several doctors and specialists. However, being both a homeopath and a doctor, I have discovered that I have been the first person to see the complete picture. It is one disease or another, which has been overlooked, and then we do some tests in order to verify my diagnosis."

A moral aspect of this situation was explained by a male participant. “If a person experiences that there are uninteresting sides to my [the patient] story, you may want to avoid telling whatever is decisive to enable you to make a correct diagnosis according to conventional medicine.”

According to the participants, a more comprehensive toolkit was a benefit of practicing two medical systems. One MH explained:.

"That is what is so nice, that we have a toolkit with a greater variety of options. Everyone must understand that it is the best for the patients to have a therapist who master all these options. I am not negative to conventional medicine as lots of it is positive. However, it is far too much of it."

He claimed further that “homeopathy has its advantage when it comes to chronic diseases, especially when psychological events manifest themselves in the body. In such cases homeopathy is absolutely outstanding.” A pleasure of professionalism was something the MHs appreciated. One female said:

"I can only speak for myself and what it has done to me when it comes to mastering two worlds simultaneously. It is the feeling of professionalism that is simply so good. I feel twice as good as a doctor than if I had mastered only one of these worlds."

These data demonstrate that the MHs always looked for severe symptoms. Alertness and awareness were always present, to avoid ignoring serious events or to decide whether further examination was necessary. They gave patients time and space, which enabled the MHs to see the complete picture. When practicing both homeopathy and conventional medicine they felt very competent. A more comprehensive toolkit gave them a feeling of flow in their daily work.

## Discussion

This study provides novel qualitative insight into how MHs understand HA and AE, and how they evaluate risk. Based on the above we found that the MHs demonstrated relevant competence of risk in clinical practice. According to the participants, HA was a common event and a criterion for distinguishing HA from AE was a feeling of well-being, which is in accordance with the results from our previous study among classical homeopaths [29]. Moreover, the MHs stated that there was no secondary effect (a counter-reaction from the body defense mechanism, see explanation below) of homeopathic remedies and AE may be defined as aggravations without dynamics, meaning there is no progression in the patient’s symptoms. There was disagreement among the participants whether or not homeopathic treatment produced AE, which is in line with the results from our previous study [29]. Further, a disruptive reaction may produce new symptoms in the patient, allowing the practitioner to prescribe a new remedy. A more comprehensive toolkit was perceived to be an advantage when practicing two medical systems.

When the participants evaluated risk in homeopathic consultations, the doctor presented within them was always present looking for severe symptoms, indicating malicious illness, also called red flag situations. However, the classical homeopaths in our previous study also showed relevant competence of risk, based on evaluation of the patient’s symptoms according to Hering’s Law of Cure. This law claims that the symptoms proceed in reverse order from the most important organs to the least important organs, from within outwards (most central organ first) and from above downwards (from head to feet) [39].

The first author, who has worked as a homeopath for many years, was interested in the apprehension about primary and secondary effects of the remedy, as she found discrepancies between the participants’ understanding of these concepts and the homeopathic literature. It is stated in Organon paragraph 63–65 [40] that a homeopathic remedy causes a certain alteration in the health of the individual for a longer or shorter period (primary action). The symptoms produced due to this action are primary symptoms. A primary action is followed by a counter-reaction from the body defense system and control mechanism (secondary action). The symptoms produced from this reaction are secondary symptoms. Those two reactions constitute the remedy’s total impact on the body. The sum of the symptoms is the remedy’s total symptoms. We suggest that HA may be explained as secondary effects of a remedy (counter-reaction). In order to evaluate risk, we find it important for the practitioners to understand homeopathic philosophy to enable distinction between all the different concepts in homeopathy. This may increase the understanding of the direction of the homeopathic cure and patient safety.

**Figure 1:**
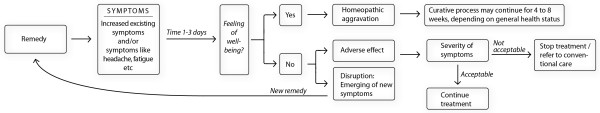
Relationship between homeopathic aggravation, adverse effects and disruptions.

Disruptions following a remedy are often found in clinical practice [39,41]. Vithoulkas states in his book Levels of Health p. 101–108 that the interpretations of such a reaction differ according to the patient’s level of health. In simple cases disruption occurs when the remedy is close but not correct and when the patient is sensitive to the remedy. This causes disappearance of some symptoms and creation of new symptoms. These new symptoms may be defined as AE in conventional medicine. If the symptoms that emerge are unclear, the homeopath should wait until he has a pattern that indicates a new remedy [39]. In more severe cases, disruptions are more likely to occur as the defense mechanism is weak and compromised. Data from this study is in line with Vithoulkas’ understanding of disruption. After having taken the remedy, the patient may experience increased symptoms or headache, fatigue or sleepiness which may last for some days. If a feeling of well-being emerges, it is classified as HA. If new symptoms appear which the homeopath recognizes as belonging to another remedy, it is classified as disruption, and the new remedy is given to the patient. Finally, if symptoms appear without a feeling of well-being, and the symptoms are not recognized, they are categorized as AE. This process is outlined in Figure 1: Relationship between homeopathic aggravation, adverse effects and disruptions.

This figure is an elaboration of a previous version [29] and shows the relationship between homeopathic aggravations, disruptions and adverse effects. This relationship is based on a comparison between the empirical results and a discussion of essential homeopathic literature.

This study revealed several advantages for the patients as the MHs looked for dangerous symptoms, which may enhance safety. According to the participants, homeopathic treatment enabled reduction of conventional medication which resulted in fewer adverse effects related to western medicine. Further, as the complete picture emerged during the consultation, unforeseen medical diagnoses were established. We argue that this practice is of great value for the patients and in line with sound professional practice as the latter is defined by professionals based on current medical evidence and professional knowledge [21]. Whether homeopathy lacks medical evidence is a matter of heated debate, as Randomized Controlled Trials (RCT) of homeopathy mostly demonstrates lack of efficacy. However, uncontrolled studies of homeopathic practice document consistent, strong therapeutic effects and sustained patient satisfaction [42]. Moreover, ethical principles of beneficence and non-maleficence may be perceived as legal standards for sound professional practice [21]. From the patients’ point of view the criteria for sound professional practice is therefore fulfilled provided that the MHs’ practice does not cause injuries.

### Methodological aspects/limitations

The study design was qualitative and explorative. This study is not designed to determine if homeopathic treatment may reduce the use of conventional medicines. Thus, it is merely a perception of the participants. No evidence is presented in this study to support that adverse effects from Western medicine will decrease. Our aim was to add new knowledge and hypotheses for further important research on patient safety of which there is little previous knowledge [43]. The participants were encouraged to speak their minds regardless of pressure from the peers [30]. However, this does not necessarily ensure an open conversation among the participants. In order to obtain validity, the quotations were sent to the participants for verification [44]. A model describing the difference between aggravations, disruptions and adverse effects was developed based on the literature review and data from the interviews and sent to the participants for comments. This was the strategy to achieve saturation and theoretical transparency [45]. Inter coding agreement was achieved as three independent researchers coded and explored the data [38,46]. The MHs were experienced in practicing both conventional medicine and homeopathy. We claim that this may be an advantage as it takes years to achieve competence in two different fields. However, MHs with less experience may hold other options about the themes in question.

### Implications for practice

We argue that these findings may have implications for practice. We found that the MHs in this study demonstrated relative competence of risk in clinical practice. However, homeopathic practice is vague and unequally regulated in many European countries, and as such associated with indirect risks. This can be controlled for by increased collaboration between primary care providers and homeopaths. In a Norwegian proposition to the Odelsting (number 27 p.82) [28], it is stated that the Central Health Authorities ought to assume greater responsibility for increasing knowledge about complementary medicine. This knowledge may form the basis for collaboration between CAM practitioners and health care providers. So far this has been poorly recorded.

Findings from this study are transferable to other health care professionals both inside and outside the conventional health care system. It is important to develop awareness in order to always look for severe symptoms, to avoid ignoring serious events and to decide whether further examination is necessary. Furthermore, it may be essential for patient safety to give the patients time and space, which enables the practitioner to see the complete picture. In additional this may give the practitioner an extended feeling of professionalism.

This study reveals discrepancies between the MHs’ understanding of different homeopathic concepts and theory. In further research we find it important to improve and develop concepts that are unique to homeopathy, in order to validate and modernize this medical practice. This is also a means to improve the assessment of risk and patient safety.

## Conclusion

This explorative study investigated how Medical Homeopaths understood and assessed risk in their clinical practice. A feeling of well-being emerging soon after taking the remedy was the most important criterion for discriminating between Homeopathic Aggravations and Adverse Effects in clinical practice. The Medical Homeopaths used the view of both professions and always looked for red flag situations in the consultation room. They combined knowledge from two treatment systems which may have advantages for the patient. These tentative results deserve further research efforts to improve patient safety among users of homeopathy.

## Abbreviations

AE: Adverse Effects; CAM: Complementary and Alternative Medicine; GP: General Practitioner; HA: Homeopathic Aggravation; MH: Medical Homeopath; RCT: Randomized Controlled Trials.

## Competing interests

The authors declare that they have no competing interests.

## Authors’ contributions

TR conceived the study, developed the interview guide and the qualitative methodology, was the moderator of the interviews, transcribed the data, analyzed and coded the data and drafted the manuscript. TA: observed the interviews, analyzed and coded the data, reviewed subsequent version of the manuscript. AS: Observed one interview, developed the interview guide and the qualitative methodology, analyzed and coded the data, reviewed subsequent version of the manuscript. All authors read and approved the final manuscript.

## Pre-publication history

The pre-publication history for this paper can be accessed here:

http://www.biomedcentral.com/1472-6882/12/150/prepub

## Supplementary Material

Additional file 1 Focus group interview with medical homeopaths concerning homeopathy and risk. Click here for file

Additional file 2 **Below are some definitions of essential terms that will be used in the interview.** Please read them so that we have the same understanding of the terms when we start the discussion. Click here for file
