# Supplementary material for: The Red flag! risk assessment among medical homeopaths in Norway: a qualitative study
Source: BMC Complement Altern Med. 2012 Sep 11;12:150. doi: 10.1186/1472-6882-12-150 (PMC3488491; doi:10.1186/1472-6882-12-150)
Supplement: Additional file 1 — Focus group interview with medical homeopaths concerning homeopathy and risk. [file 1472-6882-12-150-S1.docx]

***Additional file I***

**Focus group interview with medical homeopaths concerning homeopathy and risk**

**Interview guide**

**Introduction**

We would like to find out about your experience with **homeopathic aggravation**. We would like you to tell us about your experiences with this phenomenon from your own practice. We will also discuss whether homeopathic treatment causes **adverse effects**. Moreover, we will discuss other aspects of risk, such as **”doctor delayed contact”, whether patients who quit seeking conventional medical care represent a risk**, **interaction** between conventional medicine and homeopathic remedies/herbs, and your experience treating **sensitive persons**. You have been chosen for this interview because you have experience from clinical practice as doctors and homeopaths and thus hold a unique competence. We would therefore like to discuss with you **how you experience belonging to two medical paradigms of such a different nature**.

In a **focus group interview** there are no incorrect answers. On the contrary, we look for different points of view and experiences with the themes in question. Please feel free to say what you want even though your opinion may differ from those of the others. We are interested in all comments and please remember that we are interested in everyone’s opinion.

**1. Discussion guidelines**

Before we start, let me make some suggestions that will hopefully give us a more productive discussion. I encourage you to feel free to speak, but only one person at a time. We will be tape recording this discussion, as we don’t want to miss any of the information. In this interview we will use each other’s first names. The article to be written at a later stage will not state your names or any information that may reveal your identity. This is a means to secure confidentiality.

My role is to ask questions and listen. I will not participate in the dialogue, but I would like you to speak openly. I will ask you some questions and lead the discussion from question to question. Often in a group like this some people talk a lot whereas others talk less. However, today it is important that everyone has a chance to share their various experiences. If some of you have a lot to say, I ask that you also give room for the others to speak. If you don’t have a lot to say, I will call on you directly to obtain your opinion.

**2. Why is this research important?**

Why is this research important? Because it has been called for by the professionals (British Journal of Homeopathy). Moreover, it will contribute to ensuring the quality of homeopathy. In addition, it is also a means of developing the field and promotes a small group of Norwegian therapists holding a unique competence in two different fields. Research is also important for the patients’ safety. For the authorities and other health workers it enables them to give information about homeopathic treatment which is based on research. NHL (Norwegian Association of Homeopaths) also think this research is important, and has therefore supported this project by NOK 30,000.

**3. Presentation of the participants**

**4. Presentation of co-researchers in the project**

**5. The initial question**

I will start by asking a question about homeopathic aggravations.

**Homeopathic aggravations**

- Do you experience in your practice that your patients report homeopathic aggravations. If yes, how often?
- Do you inform your patients that this may occur? If yes, how do you inform them?

**Key questions**

- How do you determine whether the aggravations your patient tells you about are homeopathic aggravations and not a natural worsening caused by the disease itself?
- Are there any changes that the patients tell you about that may indicate in which direction this will lead? Towards improvement or continuous worsening.
- ”Doctor delayed contact”. Patients who use homeopathy as some of them see their doctor at a later stage than they normally would, and as a result the homeopathic treatment may represent a health risk.
- Have you ever stopped the homeopathic treatment and proceeded with conventional treatment?
- Have you ever been consulted by other alternative therapists for medical advice regarding patients they are treating?

**Adverse effects**

- Have you had patients reporting adverse effects from homeopathic medication?
- If yes, what do you do? What are your routines?

**Key symptoms**

- All of us know how difficult it may be to find the correct homeopathic remedy and quite often the patient may need more than one remedy before the disease is cured. Have you experienced that your patients have had adverse effects from the treatment when you have prescribed
- An incorrect homeopathic remedy/potency or time interval?
- Discontinuation of conventional treatment, or by interaction between conventional medication and homeopathic medication.
- ”Highly sensitive persons” is known from the literature. Which experiences do you have with this kind of patients and how do you treat them?
- How do you determine whether the symptoms experienced by the patients following homeopathic treatment are a part of the healing process/homeopathic aggravations or possible adverse effects?
- Based on our discussion till now, do you see any criteria which may separate possible adverse effects from homeopathic aggravations?

**Patients who stop using conventional medication**

- Some patients stop using conventional medication completely. What do you think about that?
- Do you consider discontinuation of conventional treatment a health risk?
- Why do you think patients choose to see you, being both a doctor and a homeopath?

**How is it belonging to two medical paradigms?**

- How would you describe the conventional and complimentary medical paradigms?
- How do you merge these two philosophies in your practice?
- What does it do to you as a professional?
- How do your patients benefit from this?
- Are there any disadvantages for you in your practice to hold competence in both of these paradigms? (How do other doctors view a doctor who is also a homeopath, also how do homeopaths view a homeopath who is also a doctor?)

**Communiation**

- Many patients wish to speak to their therapist about their problems of existential nature which are often connected to severe diseases. How do you do this?
- How may homeopathic interviews contribute to opening up for such communication?
- Is it possible that lack of such communication may represent a health risk for the patients? If yes, how and why?

**Closing questions**

- What do you consider the most important issues that we have discussed today?

**Closing**

- Co-researcher, will you please give us your closing remarks on the most important issues that we have discussed today (two to three minutes).
