# Supplementary material for: The Red flag! risk assessment among medical homeopaths in Norway: a qualitative study
Source: BMC Complement Altern Med. 2012 Sep 11;12:150. doi: 10.1186/1472-6882-12-150 (PMC3488491; doi:10.1186/1472-6882-12-150)
Supplement: Additional file 2 — Below are some definitions of essential terms that will be used in the interview. Please read them so that we have the same understanding of the terms when we start the discussion. [file 1472-6882-12-150-S2.docx]

***Additional file II***

**Definitions**

Below are some definitions of essential terms that will be used in the interview. Please read them so that we have the same understanding of the terms when we start the discussion.

**Homeopathic aggravation:** Homeopathic aggravation is a “temporarily worsening of existing symptoms following the administration of a correctly chosen homeopathic prescription, which indicates a favorable response to treatment”. Patients usually experience such events soon after the administration of the medicine or up to 14 days later [[7](#_ENREF_7)]

**Adverse effects**

This definition is based on the National Medicines Agency: Adverse Effects (AE) include all diseases or unwanted and/or harmful reactions appearing during the study period, whether or not they may be connected to the actual treatment. In order to decide if there is a correlation between the treatment and the reactions we will use the classification described below.

1. Probable

There is a correlation in time between the reaction and the treatment. The reaction cannot be explained by the patient’s clinical status. The reaction is already well-known or may be expected. The reaction appears after repeating the remedy or disappears after a remedy break.

1. Possible

There is a correlation in time between the reaction and the treatment. The reaction is already well-known or may be expected. Other factors may explain the reactions.

1. Not likely

There is a correlation in time between the reaction and the treatment. No correlation between appearances of reactions after repeating the remedy or disappearance of reactions after a remedy break. The reactions may be explained by several other factors.

1. No correlation

There is no correlation in time between the reaction and the treatment. The reaction is most likely caused by other factors, for example symptoms from another parallel illness.

1. Impossible to assess

It is impossible to assess any correlation between the reaction and the treatment due to the amount and content of the data.
